# White Matter Hyperintensities as a Risk Factor for Ischemic Stroke in Patients With Systemic Lupus Erythematosus

**DOI:** 10.3389/fneur.2021.738173

**Published:** 2021-12-13

**Authors:** Eri Sano, Shigeki Arawaka

**Affiliations:** Division of Neurology, Department of Internal Medicine IV, Faculty of Medicine, Osaka Medical and Pharmaceutical University, Takatsuki, Japan

**Keywords:** systemic lupus erythematosus, white matter hyperintensity, ischemic stroke, risk factor, epidemiology

## Abstract

**Objective:** The occurrence of ischemic stroke in patients with systemic lupus erythematosus (SLE) can cause extended periods of reduced daily activities. However, the risk factors for ischemic stroke in SLE patients are not fully elucidated. Herein, we examined the effect of white matter hyperintensities (WMH) on the occurrence of ischemic stroke in SLE patients.

**Methods:** We analyzed the relationship between WMH burden and ischemic stroke using follow-up brain magnetic resonance imaging (MRI) data of 79 patients with SLE. Of these patients, 16 developed stroke during the observation period. WMH on MRI were classified into periventricular hyperintensities and deep white matter hyperintensities (DWMH), while the lesion extent was graded using the Fazekas scale.

**Results:** Kaplan–Meier curves showed that ischemic stroke events were significantly associated with age at initial brain MRI of ≥40 years (*p* = 0.015) and history of anti-phospholipid syndrome (*p* = 0.030). Additionally, ischemic stroke events were significantly associated with a one grade deterioration of periventricular hyperintensities (*p* = 0.003) and a one grade deterioration of DWMH (*p* = 0.002). Multivariate analysis using the logistic regression model showed that a one grade deterioration of DWMH was an independent risk factor for ischemic stroke (hazard ratio, 6.0; 95% confidence interval, 1.3–27.4).

**Conclusions:** Although several factors affect the occurrence of ischemic stroke, SLE patients show increased risk of ischemic stroke via development of DWMH. An observation of DWMH deterioration on follow-up brain MRI may be useful for assessing the risk of ischemic stroke in SLE patients.

## Introduction

Systemic lupus erythematosus (SLE) is an autoimmune disease characterized by the production of autoantibodies, immune complex deposition, and multiple target organ damage (1). The occurrence of SLE varies considerably worldwide (2). In Japan, the prevalence of SLE ranges from 3.7 to 37.7 per 100,000 people. SLE mainly affects women in the 20–40-year age group (3, 4). In an international cohort study, 40.3% of SLE patients showed neuropsychiatric symptoms during a mean observation period of 1.9 ± 1.2 years (5). Neurological manifestations of SLE include headache, cognitive impairment, myelopathy, and peripheral neuropathy (6). Additionally, cerebrovascular diseases often occur in SLE patients and cause a range of disturbances in daily living (7). For example, the risk of cerebral infarction in adult SLE patients was 2.18-fold higher than that in healthy individuals (8). However, the risk factors for occurrence of ischemic stroke in SLE patients are poorly characterized.

In the general population, white matter hyperintensities (WMH) are a common finding in brain magnetic resonance imaging (MRI) of adults ≥50 years old (9). Furthermore, people ≥65 years old show deterioration of WHM. The occurrence of WMH is correlated with aging, while vascular factors including hypertension, smoking, and diabetes mellitus can increase the risk for WMH (9). The presence of WMH also increases the risk of cerebrovascular diseases and cognitive impairment in the general population (10). A meta-analysis of prospective longitudinal studies in the general population reported that WMH on brain MRI increased the risk of ischemic stroke [hazard ratio (HR), 3.1; 95% confidence interval (95%CI), 2.3–4.1] (11). Although WMH are the most common brain MRI findings in SLE patients (12, 13), the influence of WMH on the occurrence of ischemic stroke in SLE patients remains unclear. In the present study, we investigated whether WMH were a risk factor for the occurrence of ischemic stroke in SLE patients.

## Methods

### Patients

We analyzed 79 patients with SLE who visited Osaka Medical and Pharmaceutical University Hospital and underwent repeated brain MRI between April 2012 and June 2018. We enrolled patients with (1) age of >16 years, (2) a diagnosis according to the 1997 revised American College of Rheumatology classification criteria for SLE (14), and (3) no obvious evidence of any other disease that might explain the SLE-like symptoms. In the criteria, patients were required for fulfilling four or more criteria among clinical and immunological disorders. We excluded patients with (1) abnormalities on brain MRI (e.g., hydrocephalus, neoplasm, or metal deposition) or (2) history of brain trauma or operation. Only women were included in this study because the majority of patients at our facility were women. All patients had received an initial brain MRI for screening of brain lesions or for evaluating the cause of neuropsychiatric symptoms, and all patients had received more than one brain MRI in total (twice in 39 patients, three times in 11 patients, five times in seven patients, six times in five patients, seven times in four patients, and >10 times in four patients). A follow-up brain MRI was performed to evaluate the status of cerebrovascular diseases and other central nervous system lesions.

For diagnosis of anti-phospholipid syndrome (APS), we confirmed positivity for anti-phospholipid antibodies on two or more occasions that were at least 12 weeks apart and the presence of clinical histories (e.g., recurrent spontaneous abortion or thrombosis) (15). We defined positivity for antiphospholipid autoantibody as either anti-cardiolipin antibodies of ≥10.0 U/ml, anti-cardiolipin-β2-glycoprotein complex antibodies of ≥3.5 U/ml, or a lupus anticoagulant of ≥1.3. Clinical data were retrospectively retrieved from medical records. This study was conducted according to the 2013 Helsinki Declaration. The study protocol was approved by Osaka Medical and Pharmaceutical University Ethics Committee. The requirement for informed consent was waived because of the retrospective nature of the study and removal of all individual patient identifiers from the data (Approval number # 609).

### Clinical Findings and Laboratory Examinations

We collected baseline information, including age at SLE diagnosis and initial brain MRI, sex, vascular factors (history of smoking, diabetes mellitus, hypertension, and dyslipidemia), autoimmune abnormality (history of APS), and anti-platelet or anti-coagulant drug treatment for all patients. Smoking status was evaluated by interviews, and the patients were classified as current and past smokers or nonsmokers. Hypertension was defined with a resting blood pressure of ≥140/90 mmHg or current use of antihypertensive agents (16). Diabetes mellitus was judged according to the American Diabetes Association criteria or by current medication history (17). Dyslipidemia was defined as low-density lipoprotein cholesterol levels of ≥160 mg/dl, triglyceride levels of ≥150 mg/dl, or by current medication history (18). Additionally, we collected baseline information on the titers of anti-nuclear antibodies (ANA), anti-double-stranded DNA antibodies, and the Systemic Lupus Erythematosus Disease Activity Index in measurable patients (19). The titers of ANA and anti-double-stranded DNA antibodies were measured by enzyme-linked immunosorbent assay.

### Assessment of WMH and Ischemic Stroke on Brain MRI

Diffusion, T2, FLAIR, and T2^*^-weighted sequences (slice thickness, 2 mm) were obtained using a 3.0T MRI (Signa HDxt, GE healthcare). To analyze WMH, the grades of periventricular hyperintensities (PVH) and deep WMH (DWMH) were evaluated using the Fazekas rating scale (20). PVH were evaluated on FLAIR-weighted transverse images covering the anterior horn and body of the lateral ventricles. DWMH were evaluated on FLAIR-weighted transverse images covering the semioval center. Initial brain MRI was used as the baseline image. To assess temporal changes in WMH, we evaluated the differences in the grades of PVH and DWMH by subtracting the grades between baseline and the most recent MRI images. These data indicated a one grade deterioration of PVH and DWMH. Ischemic stroke was defined as acute onset of focal neurological deficits with abnormal high signal intensities on diffusion-weighted images and low apparent diffusion coefficient values on the apparent diffusion coefficient map.

### Statistical Analysis

We analyzed the association between candidate factors and an ischemic stroke event from the initial brain MRI to the most recent MRI using Kaplan–Meier curves and the log-rank test. We estimated the HRs of the candidate factors for ischemic stroke by univariate analysis using the Cox proportional hazards model. Candidate factors were then subjected to multivariate analysis using the logistic regression model. Continuous values are expressed as median and interquartile range (IQR). A *p*-value <0.05 was considered statistically significant. All statistical analyses were performed using statistical software (JMP Pro v14; SAS Institute Inc., Cary, NC, USA).

## Results

### Patient Characteristics

We used clinical and laboratory data from 79 patients with SLE who underwent repeated brain MRI (Table 1). All patients were women. The median age at diagnosis was 31 years (IQR, 20–44 years), while the median age at initial brain MRI was 40 years (IQR, 31–53 years). Of these patients, 21 had APS (26.6%) and 16 patients (20.3%) had ischemic stroke as a new event after initial brain MRI. The median interval from initial brain MRI to the onset of ischemic stroke was 2 years (IQR, 2–10 years). At initial brain MRI, PVH were observed in 23 patients (29.1%) and DWMH were observed in 30 patients (38.0%). In follow-up brain MRI, a one grade deterioration of PVH and DWMH on the Fazekas scale was observed in 19 (24.1%) and 24 (30.4%) patients, respectively.

**Table 1:** Demographic data of 79 patients with Systemic Lupus Erythematous (SLE).

	***n* (%)**	**Median [Interquartile range]**
Female	79 (100)	
Age at diagnosis of SLE		31 [20–44]^a^
Age at initial brain MRI		40 [31–53]^a^
Occurrence of ischemic stroke	16 (20.3)	
APS	21 (26.6)	
Current and past smoker	15 (19.0)	
Hypertension	32 (40.5)	
Diabetes mellitus	15 (19.0)	
Dyslipidemia	12 (15.2)	
Anti-platelet drug treatment	25 (31.6)	
Anti-coagulant drug treatment	4 (5)	
Titers of ANA		480 [160–1280]^b^
Titers of anti-double-stranded DNA antibodies		6.9 [2.8–47.9]^c^
SLEDAI		11.5 [6–19.3]^d^
PVH	23 (29.1)	
DWMH	30 (38.0)	
One grade deterioration of PVH	19 (24.1)	
One grade deterioration of DWMH	24 (30.4)	

a *n = 79*;

b *n = 36*;

c *n = 45*;

d *n = 30*.

*ANA, anti-nuclear antibodies; APS, antiphospholipid syndrome; DWMH, deep white matter hyperintensities; MRI, magnetic resonance imaging; PVH, periventricular hyperintensities; SLE, systemic lupus erythematosus; SLEDAI, systemic lupus erythematosus disease activity index*.

### Assessment of Clinical Factors Associated With Ischemic Stroke in Patients With SLE

**Figure 1:**
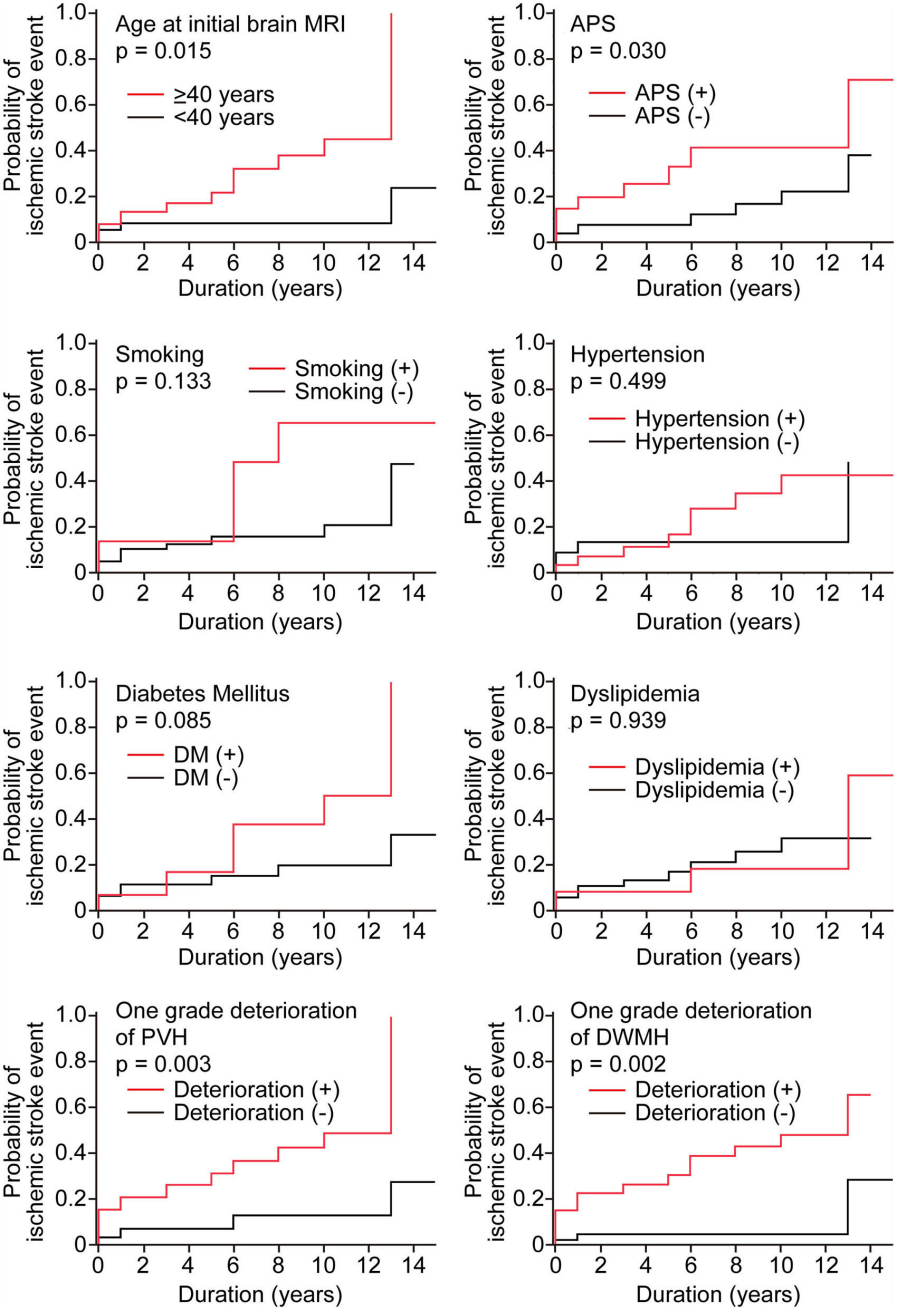
Kaplan–Meier curves of ischemic stroke events in the study population disaggregated by the presence or absence of candidate risk factors. Kaplan–Meier curves were analyzed with the log-rank test. Duration represents the observation period from initial brain MRI to the most recent MRI. The red line shows the group with candidate factors, and the black line shows the group without candidate factors. In age, patients were classified into two groups <40 years (red line) and ≥40 years (black line).

To explore the candidate factors associated with ischemic stroke in patients with SLE, we analyzed differences in the occurrence of ischemic stroke between patients with and without each factor using Kaplan–Meier curves with the log-rank test. We were unable to analyze the immunological data (e.g., the titers of ANA and anti-double-stranded DNA antibodies) or the Systemic Lupus Erythematosus Disease Activity Index scores because the number of measurable patients was small. Kaplan–Meier curves showed that ischemic stroke events were significantly associated with age at initial brain MRI of ≥40 years (*p* = 0.015) and history of APS (*p* = 0.03) (Figure 1). Additionally, ischemic stroke events were significantly associated with a one grade deterioration of PVH (*p* = 0.003) and a one grade deterioration of DWMH (*p* = 0.002). Vascular factors, including history of smoking, diabetes mellitus, hypertension, and dyslipidemia, showed no association with the ischemic stroke events in our SLE patients.

### Assessment of Clinical Factors Predictive for Ischemic Stroke in Patients With SLE

To determine the predictive factors for the occurrence of ischemic stroke in patients with SLE, we analyzed clinical factors by univariate analysis using the Cox proportional hazards model (Table 2). The ischemic stroke events were associated with age at initial brain MRI (HR, 3.9; 95%CI, 1.2–12.9) and history of APS (HR, 2.7; 95%CI, 1.04–7.40). Furthermore, the ischemic stroke events were associated with a one grade deterioration of PVH (HR, 4.2; 95%CI, 1.5–12.1) and a one grade deterioration of DWMH (HR, 6.0; 95%CI, 1.7–21.3). Vascular factors, including history of smoking, diabetes mellitus, hypertension, and dyslipidemia, showed no significant association with the ischemic stroke events in our patients with SLE. Assessment of candidate factors by multivariate analysis using the logistic regression model showed that ischemic stroke events were only associated with a one grade deterioration of DWMH (HR, 6.0; 95% CI, 1.3–27.4) (Table 2). These findings suggested that a one grade deterioration of DWMH was an independent risk factor for predicting ischemic stroke appearance in patients with SLE.

**Table 2:** Comparison of clinical factors predictive for ischemic stroke in patients with SLE.

	**Univariate analysis**	**Multivariate analysis**
	**HR (95% CI)**	**HR (95% CI)**
Age at initial brain MRI	3.9 (1.2–12.9)	2.9 (0.6–13.5)
APS	2.7 (1.0–7.4)	2.2 (0.6–7.3)
Current and past smoker	2.2 (0.8–6.3)	2.1 (0.6–7.5)
Hypertension	1.4 (0.5–3.7)	0.4 (0.1–1.7)
Diabetes mellitus	2.3 (0.8–6.5)	1.8 (0.5–7.0)
Dyslipidemia	0.9 (0.3–3.1)	0.7 (0.2–2.7)
One grade deterioration of PVH	4.2 (1.5–12.1)	1.2 (0.3–5.1)
One grade deterioration of DWMH	6.0 (1.7–21.3)	6.0 (1.3–27.4)

*APS, antiphospholipid syndrome; CI, confidence interval; DWMH, deep white matter hyperintensities; HR, hazard ratio; MRI, magnetic resonance imaging; PVH, periventricular hyperintensities*.

*Hazard ratios were analyzed by univariate analysis using the Cox proportional hazards model or multivariate analysis using the logistic regression model*.

## Discussion

In the present study, we analyzed the risk factors for ischemic stroke in SLE patients to evaluate the influence of WMH burden on clinical events in the brain. In multivariate analysis using the logistic regression model, we identified a one grade deterioration of DWMH as an independent risk factor for the occurrence of ischemic stroke in patients with SLE. By contrast, a history of smoking, hypertension, diabetes mellitus, and dyslipidemia were not independent risk factors for this event. In the general population, these vascular factors are established as independent factors for ischemic stroke (21). The cause of this disparity between our patients with SLE and the general population is unclear. Previous study has demonstrated that hypertension is an independent risk factor for PVH and DWMH in patients with SLE (22). Thus, vascular factors may contribute to the occurrence of ischemic stroke via generation of WMH in patients with SLE, while the WMH burden may contribute to the occurrence of ischemic stroke. Our findings also suggest that therapeutic interventions designed to delay DWMH progression may prevent the occurrence of ischemic stroke in patients with SLE. Follow-up brain MRI to monitor DWMH deterioration may be useful for assessing ischemic stroke risk in patients with SLE.

Anti-phospholipid syndrome (APS) is often associated with SLE and is known to promote thrombosis in the cerebral arterial system, resulting in ischemic stroke and transient ischemic attack (23). However, in the present study, APS was not an independent risk factor for ischemic stroke in patients with SLE. The majority of our patients with APS had experienced an ischemic stroke before their initial MRI. Additionally, anti-platelet and/or anti-coagulant therapy had already been initiated in these patients. This preventive therapy may have reduced the risk of ischemic stroke during the observation period in patients with APS.

WMH are one of the main MRI features of small vessel disease in addition to acute lacunar infarcts or hemorrhages, lacunes as fluid-filled cavities of old infarcts, visible perivascular spaces, microbleeds, and brain atrophy (24). These changes are caused by variable etiologies, including arteriolosclerosis, cerebral amyloid angiopathy, and inflammatory and immunological causes (25). Pathologically, WMH are characterized by demyelination, astrogliosis, spondylosis, axonal loss, and widened perivascular spaces (10). It is difficult to address the etiology of WMH in SLE, although vascular factors are shown to be associated with WMH in SLE (22). To address this issue, further studies are required with detailed laboratory data (e.g., oligoclonal bands in the cerebrospinal fluid of SLE patients) and pathological examination of SLE brains.

In the present study, we examined changes in the brains of SLE patients using the same MRI conditions, which allowed us to assess WMH deterioration at follow-up brain MRI. However, there were some limitations in our study. First, this was a retrospective study. Second, the sample size of patients followed-up for analysis of ischemic stroke was small. Third, we were unable to assess the contribution of immunological factors to ischemic stroke because of a partial lack of immunological data. Finally, it is unclear whether the extent of WMH on the Fazekas scale is associated with ischemic stroke. Prospective large multicenter cohort studies are required to validate the contribution of WMH to the occurrence of ischemic stroke.

## Conclusions

Patients with SLE have increased risk of ischemic stroke via development of DWMH. Follow-up brain MRI to monitor DWMH deterioration may be useful for assessing the risk of ischemic stroke.

## Data Availability Statement

The original contributions presented in the study are included in the article, further inquiries can be directed to the corresponding author.

## Ethics Statement

The studies involving human participants were reviewed and approved by Osaka Medical and Pharmaceutical University Ethics Committee. Written informed consent for participation was not required for this study in accordance with the national legislation and the institutional requirements.

## Author Contributions

All authors listed have made substantial, direct, and intellectual contribution to the work and approved it for publication.

## Conflict of Interest

The authors declare that the research was conducted in the absence of any commercial or financial relationships that could be construed as a potential conflict of interest.

## Publisher's Note

All claims expressed in this article are solely those of the authors and do not necessarily represent those of their affiliated organizations, or those of the publisher, the editors and the reviewers. Any product that may be evaluated in this article, or claim that may be made by its manufacturer, is not guaranteed or endorsed by the publisher.

## Abbreviations

ANA: anti-nuclear antibodies
APS: anti-phospholipid syndrome
DWMH: deep white matter hyperintensities
HR: hazard ratio
IQR: interquartile range
MRI: magnetic resonance imaging
PVH: periventricular hyperintensities
SLE: systemic lupus erythematosus
WMH: white matter hyperintensities.
